# Fano-Rashba effect in thermoelectricity of a double quantum dot molecular junction

**DOI:** 10.1186/1556-276X-6-618

**Published:** 2011-12-07

**Authors:** YS Liu, XK Hong, JF Feng, XF Yang

**Affiliations:** 1 Jiangsu Laboratory of Advanced Functional materials and College of Physics and Engineering, Changshu Institute of Technology, Changshu 215500, China; 2 Department of Theoretical Chemistry, School of Biotechnology, Royal Institute of Technology, S-106 91 Stockholm, Sweden

**Keywords:** Rashba spin-orbit interaction, Aharonov-Bohm interferometer, Quantum dots, Fano effects

## Abstract

We examine the relation between the phase-coherent processes and spin-dependent thermoelectric effects in an Aharonov-Bohm (AB) interferometer with a Rashba quantum dot (QD) in each of its arm by using the Green's function formalism and equation of motion (EOM) technique. Due to the interplay between quantum destructive interference and Rashba spin-orbit interaction (RSOI) in each QD, an asymmetrical transmission node splits into two spin-dependent asymmetrical transmission nodes in the transmission spectrum and, as a consequence, results in the enhancement of the spin-dependent thermoelectric effects near the spin-dependent asymmetrical transmission nodes. We also examine the evolution of spin-dependent thermoelectric effects from a symmetrical parallel geometry to a configuration in series. It is found that the spin-dependent thermoelectric effects can be enhanced by controlling the dot-electrode coupling strength. The simple analytical expressions are also derived to support our numerical results.

PACS numbers: 73.63.Kv; 71.70.Ej; 72.20.Pa

## Introduction

With the fast development and improvement of experimental techniques [1-9], much important physical properties in QD molecules such as electronic structures, electronic transport, and thermoelectric effects et al have widely attracted academic attention [10-29]. QDs can be realized by etching a two-dimensional electron gas (2DEG) below the surface of AlGaAs/GaAs heterostructures or by an electrostatic potential. Confinement of particles in all three spatial directions results in the discrete energy levels such like an atom or a molecule. We can therefore think of QDs as artificial atoms or molecules. The small sizes of QDs make the phase-coherent of waves become more important, and quantum interference phenomena emerge when the particles moves along different transport paths. Fano resonances, known in the atomic physics, arise from quantum interference effects between resonant and nonresonant processes [30]. The main embodying of the Fano resonances is the asymmetric line profile in the transmission spectrum, which originates from the coexistence the resonant transmission peak and the resonant transmission dip. The first experiment observation of the asymmetrical Fano line shape in the QD system has been reported in a single-electron transistor [31].

The RSOI in the QD can be introduced by an asymmetrical-interface electric field applied to the semiconductor heterostructures [32,33]. Electron spin, the intrinsic properties of electrons, become more important when electrons transport through the AB interferometer. The RSOI can couple the spin degree of freedom to its orbital motion, which provides a possible method to control the spin of transport electrons. A spin transistor by using the RSOI in a semiconductor sandwiched between two ferromagnetic electrodes has been proposed [34]. In spin Hall devices, spin-up and spin-down electrons flow in an opposite direction using the Rashba SOI and a longitudinal electric field such that the spin polarization becomes infinity [35-37]. Some theoretical and experimental works have also shown that the spin-polarization of current based on the RSOI can reach as high as 100%[38,39] or infinite [40].

Recently, an experimental measurement of the spin Seebeck effect (the conversion of heat to spin polarization) by detecting the redistribution of spins along the length of a sample of permalloy (NiFe) induced by a temperature gradient was firstly demonstrated [41]. The new heat-to-electron spin discovery can be named as "thermo-spintronics". More recently, the spin Seebeck effect was also observed in a ferromagnetic semiconductor GaMnAs [42]. Much academic work on spin-dependent thermoelectric effects in single QD attached to ferromagnetic leads with collinear magnetic moments or noncollinear magnetic moments has been reported [43-46]. Up to now, we note that most of the spin Seebeck effects are obtained by using ferromagnetic materials such as ferromagnetic thin films, ferromagnetic semiconductors, or ferromagnetic electrodes et al. In our previous work, a pure spin generator consisting of a Rashba quantum dot molecule sandwiched between two non-ferromagnetic electrodes via RSOI instead of ferromagnetic materials has been proposed by the coaction of the magnetic flux [24]. It should be noted that charge thermopower of QD molecular junctions in the Kondo regime and the Coulomb blockade regime have been widely investigated [25-29].

In the present work, we investigate the spin-dependent thermoelectric effects of parallel-coupled double quantum dots embedded in an AB interferometer, in which the RSOI in each QD is considered by introducing a spin-dependent phase factor in the linewidth matrix elements. Due to the quantum destructive interference, an asymmetrical transmission node can be observed in the transmission spectrum in the absence of the RSOI. Using an inversion asymmetrical interface electric field, the RSOI can be introduced in the QDs. The asymmetrical transmission node splits into two spin-dependent asymmetrical transmission nodes in the transmission spectrum and, as a consequence, results in the enhancement of the spin-dependent Seebeck effects near the spin-dependent asymmetrical transmission nodes. We also examine the evolution of spin-dependent Seebeck effects from a symmetrical parallel geometry to a configuration in series. The asymmetrical couplings between QDs and non-ferromagnetic electrodes induce the enhancement of spin-dependent Seebeck effects in the vicinity of spin-dependent asymmetrical transmission nodes. Although the spin-dependent Seebeck effects in the AB interferometer have not been realized experimentally so far, our theoretical study provides a better way to enhance spin-dependent Seebeck effects in the AB interferometer in the absence of the ferromagnetic materials.

## Model and method

**Figure 1:**
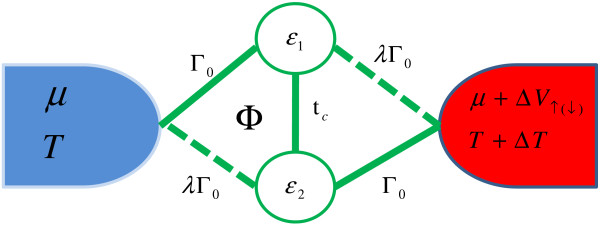
**(Color online) Schematic diagram for a thermoelectric device based on a double QD AB interferometer in the presence of magnetic flux Φ**. A spin-dependent thermoelectric voltage Δ*V_σ _*is generated when a temperature gradient Δ*T *is presented, where *μ *is the chemical potential of the metallic electrodes, and *T *is the temperature of the metallic electrode.

The schematic diagram for the quantum device based on parallel-coupled double quantum dots embedded in an AB interferometer in the present work is illustrated in Figure 1, and two noninteracting QDs embedded in the AB interferometer. QDs can be realized in the two-dimensional electron gas of an AlGaAs/GaAs heterostructure, in which a tunable tunneling barrier between the two dots is formed by using two gate voltages. So we can set *t_c _*as the coupling between the two QDs, which can be modulated by using the gate voltages [1]. The RSOI is assumed to exist inside QDs, which can produce two main effects including a spin-dependent extra phase factor in the tunnel matrix elements and interlevel spin-flip term [47,48]. In the present paper, we only consider the first term because of only one energy level in each QD. When a temperature gradient Δ*T *between the two metallic electrodes is presented, a spin-dependent thermoelectric voltage Δ*V*_↑(*↓*) _emerges. The proposed spin-dependent thermoelectric AB interferometer can be described by using the following Hamiltonian in a second-quantized form as,

(1) $$H_{total}=\underset{\alpha =L,R;\;k\sigma }{\sum }\epsilon_{\alpha k\sigma }a_{\alpha k\sigma }^{†}a_{\alpha k\sigma }+\underset{n=1,2;\sigma }{\sum }\epsilon_{n}d_{n\sigma }^{†}d_{n\sigma }-t_{c}\left(d_{1\sigma }^{†}d_{2\sigma }+H.c.\right)+\underset{k,\alpha ,\sigma ,n}{\sum }\left[V_{\alpha \sigma n}d_{n\sigma }^{†}a_{\alpha k\sigma }+H.c.\right],$$

where $a_{\alpha k\sigma }^{†}\left(a_{\alpha k\sigma }\right)$ is the creation(annihilation) operator for an electron with energy *ε_αkσ_*, momentum *k *and spin index *σ *in electrode *α*. The electrode *α *can be regarded as an independent electron and thermal reservoirs, which can be described by using the Fermi-Dirac distribution such as *f_α _*= 1/{*exp*[(*ε *- *μ_α_*)/(*k_B_T_α_*) + 1}. Here *k_B _*is the Boltzmann constant. $d_{n\sigma }^{†}\left(d_{n\sigma }\right)$ creates (destroys) an electron with energy *ε_n _*and spin index *σ *in the nth QD. *t_c _*describes the tunnel coupling between the two QDs, which can be controlled by using the voltages applied to the gate electrodes [1]. The tunnel matrix element *V_ασn _*in a symmetric gauge is assumed to be independent of momentum *k*, and it can be written as $V_{L\sigma 1}=\;|V_{L\sigma 1}|e^{i\left(\phi -\sigma \varphi_{R}\right)∕4}$, $V_{L\sigma 2}=\;|V_{L\sigma 2}|e^{-i\left(\phi -\sigma \varphi_{R}\right)∕4}$, $V_{R\sigma 1}=\;|V_{R\sigma 1}|e^{-i\left(\phi -\sigma \varphi_{R}\right)∕4}$, $V_{R\sigma 2}=\;|V_{R\sigma 2}|e^{i\left(\phi -\sigma \varphi_{R}\right)∕4}$, with the AB phase *ϕ *= 2*π*Φ/Φ_0 _and the flux quantum Φ_0 _= *h/e*. Φ can be calculated by the equation $\phi =\vec{B}\cdot \vec{S}$, where *B *is the magnetic field threading the AB interferometer and *S *is the corresponding area of the quantum ring consisting of the double quantum dots and metallic electrodes. The value S may be obtained in the previous well-known experimental work [1]. So the magnitude of the magnetic field B is 16.4*mT *when *ϕ *= 2*π*. In the absence of the RSOI, the work will come back to the previous work [24], in which a 2*π*-periodic linear conductance is obtained, and it is in good agreement with the experimental work [1]. *φ_R _*denotes the difference between *φ*_*R*1 _and *φ*_*R*2_, where *φ_Ri _*is the phase factor induced by the RSOI inside the ith QD.

In the steady state, using the Green's functions and Dyson's equations, the electric current with spin index *σ *through the AB interferometer can be calculated by [49],

(2) $$I_{\sigma }\left(\mu_{L},\;T_{L};\mu_{R},\;T_{R}\right)=\frac{e}{h}\int d\varepsilon \tau_{\sigma }\left(\varepsilon \right)\left[f_{L}\left(\varepsilon \right)-f_{R}\left(\varepsilon \right)\right],$$

and the thermal current with spin index *σ *from the electrode *α *is calculated by [50],

(3) $$J_{\sigma }^{\alpha }=\frac{1}{h}\int d\varepsilon \left(\varepsilon -\mu_{\alpha }\right)\tau_{\sigma }\left(\varepsilon \right)\left[f_{L}\left(\varepsilon \right)-f_{R}\left(\varepsilon \right)\right],$$

where *τ_σ_*(*ε*) is the transmission probability of electron with spin index *σ*, which can be given by $\tau_{\sigma }\left(\varepsilon \right)=Tr\left[\Gamma_{\sigma }^{L}G_{\sigma }^{r}\Gamma_{\sigma }^{R}G_{\sigma }^{a}\right]$. The spin-dependent linewidth matrix $\Gamma_{\sigma }^{L\left(R\right)}$ describes the tunnel coupling of the two QDs to the left (right) metallic electrode, which can be expressed as,

(4) $$\Gamma_{\sigma }^{L\left(R\right)}\left(\varepsilon \right)=\left(\begin{array}{cc} \Gamma_{11}^{L\left(R\right)} & \sqrt{\Gamma_{11}^{L\left(R\right)}\Gamma_{22}^{L\left(R\right)}}e^{+\left(-\right)i\phi_{\sigma }∕2}\\ \sqrt{\Gamma_{11}^{L\left(R\right)}\Gamma_{22}^{L\left(R\right)}}e^{-\left(+\right)i\phi_{\sigma }∕2} & \Gamma_{22}^{L\left(R\right)} \end{array}\right),$$

where $\Gamma_{nm}^{\alpha }=2\pi \sum_{k}|V_{\alpha \sigma n}∥V_{\alpha \sigma m}^{*}|\delta \left(\varepsilon -\varepsilon_{\alpha k\sigma }\right)$. $\;G_{\sigma }^{r}\left(\varepsilon \right)$ is the 2 *× *2 matrix of the fourier transform of retarded QD Green's function, and its matrix elements in the time space can be defined as $G_{n\sigma ,m\sigma }^{r}\left(t\right)=-i\Theta \left(t\right)<\left\{d_{n\sigma }\left(t\right),\;d_{m\sigma }^{†}\left(0\right)\right\}>$, where Θ(*t*) is the step function. The advanced dot Green's function can be obtained by the relation $G_{\sigma }^{a}\left(\varepsilon \right)={\left[G_{\sigma }^{r}\left(\varepsilon \right)\right]}^{+}$.

We consider the quantum system in the linear response regime such as an infinitesimal temperature gradient Δ*T *raised in the right metallic electrode, which will induce an infinitesimal spin-dependent thermoelectric voltage Δ*V_σ _*since the two tunneling channels related to spin are opened. We divide the tunneling current into two parts: one is from the temperature gradient Δ*T*, which is calculated by $\Delta I_{\sigma }^{T}=I_{\sigma }\left(\mu ,\;T;\mu ,\;T+\Delta T\right)$; the other is from the Seebeck effects, which can be calculated by $\Delta I_{\sigma }^{V}=I_{\sigma }\left(\mu ,\;T;\mu +e\Delta V_{\sigma },\;T\right)$. The spin-dependent Seebeck coefficient *S_σ _*can be calculated by [50],

(5) $$\Delta I_{\sigma }^{T}+\Delta I_{\sigma }^{V}=0.$$

After expanding the Fermi-Dirac distribution function to the first order in Δ*T *and Δ*V_σ_*, we obtain the spin-dependent Seebeck coefficient by *S_σ _*= Δ*V_σ_/*Δ*T *as,

(6) $$S_{\sigma }\left(\mu ,\;T\right)=-\frac{1}{eT}\frac{K_{1\sigma }\left(\mu ,T\right)}{K_{0\sigma }\left(\mu ,T\right)}.$$

where $K_{\nu \sigma }\left(\mu ,\;T\right)=\int d\varepsilon \left(-\frac{\partial f}{\partial \varepsilon }\right){\left(\varepsilon -\mu \right)}^{\nu }\tau_{\sigma }\left(\varepsilon \right)\left(\nu =0,1,2\right)$. *f *= {1 + *exp*[(*ε *- *μ*)/(*k_B_T*)]}^-1 ^denotes the zero bias fermi distribution (*μ *= *μ_L _*= *μ_R_*) and zero temperature gradient (*T *= *T_L _*= *T_R_*). The spin-dependent Seebeck effects can be measured in the experiments as the following descriptions. First, the AB interferometer based on DQD molecular junction can be realized by using a two-dimensional electron gas below the surface of an AlGaAs/GaAs heterostructure [1]. The RSOI in the QD can be introduced by using an asymmetrical-interface electric field. The temperature of the left electrode is kept at a constant, and that of the right electrode can be heated to a desired temperature by using an electric heater. So a temperature gradient can be generated in the DQD molecular junction. Second, the spin-dependent thermoelectric voltage can be measured by using the spin-detection technique involving inverse-spin-Hall effect [51,52]. Accompanying the electric charge flowing, the energy of electrons can also be carried from one metallic electrode to the other metallic electrode. In the linear response regime (*μ_L _*= *μ_R _*= *μ*), we assume that an infinitesimal temperature gradient Δ*T *is raised in the right metallic electrode, and the heat current $\Delta J_{\sigma }\left(\Delta J_{\sigma }=\Delta J_{\sigma }^{\alpha }\right)$ is divided into two parts following one from the temperature gradient $\Delta J_{\sigma }^{T}$ and the other from the Seebeck effects $\Delta J_{\sigma }^{V}$. They can be obtained by the equations $\Delta J_{\sigma }^{T}=J_{\sigma }\left(\mu ,\;T;\mu ,\;T+\Delta T\right)$ and $\Delta J_{\sigma }^{V}=J_{\sigma }\left(\mu ,\;T;\mu +e\Delta V_{\sigma },\;T\right)$. The total thermal current can be calculated by the sum of two terms as [50],

(7) $$\Delta J_{\sigma }=\Delta J_{\sigma }^{T}+\Delta J_{\sigma }^{V}.$$

The corresponding electronic thermal conductance *κ_el _*can be defined by $\kappa_{el}=\frac{\Delta J_{\sigma }}{\Delta T}$. After expanding the Fermi-Dirac distribution function to the first order in Δ*T *and Δ*V_σ _*to Eq. (7), we obtain the electronic thermal conductance from the temperature gradient,

(8) $$\kappa_{el,\sigma }^{T}\left(\mu ,\;T\right)=\frac{K_{2\sigma }}{hT},$$

and the electronic thermal conductance from the Seebeck effects,

(9) $$\kappa_{el,\sigma }^{V}\left(\mu ,\;T\right)=\frac{K_{1\sigma }eS_{\sigma }}{h}.$$

The differential conductance with spin index *σ *may be expressed as $G_{\sigma }\left(\mu ,\;T\right)=\frac{e^{2}}{h}K_{0\sigma }\left(\mu ,\;T\right)$. In the linear response regime, the charge and spin figure-of-merits (FOMs) can be defined as,

(10) $$Z_{C}T=\frac{S_{c}^{2}G_{c}T}{\sum_{\sigma }\kappa_{el,\sigma }^{T}+\sum_{\sigma }\kappa_{el,\sigma }^{V}},$$

and

(11) $$Z_{S}T=\frac{S_{S}^{2}G_{s}T}{\sum_{\sigma }\kappa_{el,\sigma }^{T}+\sum_{\sigma }\kappa_{el,\sigma }^{V}},$$

respectively, where $G_{c}=\frac{e^{2}}{h}\left[K_{0↑}\left(\mu ,\;T\right)+K_{0↓}\left(\mu ,\;T\right)\right]$ and $G_{s}=\frac{e^{2}}{h}\left[K_{0↑}\left(\mu ,\;T\right)-K_{0↓}\left(\mu ,\;T\right)\right]$. In this study, the phonon thermal conductance of the junction, which is typically limited by the QDs-electrode contact, has been ignored in the case of the poor link for phonon transport.

## Results and discussion

In the following numerical calculations, we set Г = 1*ev *as the energy unit in this paper. For simplicity, the energy levels of QDs are identical (*ε*_1 _= *ε*_2 _= 0).

**Figure 2:**
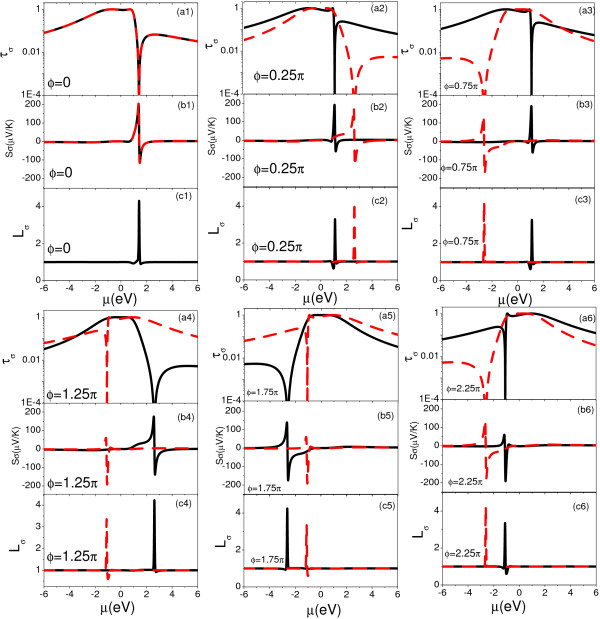
**(Color online) Spin-dependent transmission probability *τ_σ_*(logarithmic scale), spin-dependent Seebeck coefficient *S_σ_*, and spin-dependent Lorenz number *L_σ _*(in units of $L_{WF}=\frac{\pi^{2}k_{B}^{2}}{3e^{2}}$ as functions of the chemical potential *μ *under different values of *ϕ *at room temperature (T = 300 K)**. The black solid (red dashed) lines in (a n), (b n) and (c n) (*n *= 1,..., 6) represent spin-up (spin-down) transmission probability, spin-up (spin-down) Seebeck coefficient, and spin-up (spin-down) Lorenz number, respectively.

In Figure 2, we plot the spin-dependent transmission probability *τ_σ_*, spin-dependent See-beck coefficient *S_σ_*, and spin-dependent Lorenz number $L_{\sigma }=h\left(\kappa_{el,\sigma }^{T}+\kappa_{el,\sigma }^{V}\right)∕\left(e^{2}\tau_{\sigma }T\right)$ as functions of the chemical potential *μ *under several different values of *ϕ *at room temperature (T = 300 K). The phase factor *ϕ_R _*due to the RSOI inside the QD is fixed at $\frac{\pi }{2}$, which is reasonable in semiconductor heterostructures [51-54]. We first consider the case of the AB interferometer with symmetrical parallel geometry *λ *= 1 and a magnetic flux *ϕ *threading through the AB interferometer. When the interdot tunnel coupling is considered (*t_c _*= Г_0_), the transmission probability *τ_σ _*has an exact expression,

(12) $$\tau_{\sigma }=\frac{{\left(t_{c}-\mu \cos\frac{\phi_{\sigma }}{2}\right)}^{2}}{\Omega \left(\mu \right)},$$

where Ω(μ)=[(μ2-tc2)∕(2Γ0)-Γ02sin2ϕσ2]2+(μ-tc cosϕσ2)2. After a simple derivation, the transmission probability *τ_σ _*has an approximate expression as,

(13) $$\tau_{\sigma }≃\tau_{-\sigma }\left(\mu \right)+\tau_{+\sigma }\left(\mu \right),$$

where $\tau_{-\sigma }\left(\mu \right)=\frac{1}{1+q_{-\sigma }^{2}}\frac{{\left[\left(\mu -t_{c}\right)+q_{-\sigma }\Gamma_{-\sigma }\right]}^{2}}{{\left(\mu -t_{c}\right)}^{2}+\Gamma_{-\sigma }^{2}}$ and $\tau_{+\sigma }\left(\mu \right)=\frac{1}{1+q_{+\sigma }^{2}}\frac{{\left[\left(\mu +t_{c}\right)+q_{+\sigma }\Gamma_{+\sigma }\right]}^{2}}{{\left(\mu +t_{c}\right)}^{2}+\Gamma_{+\sigma }^{2}}$. The parameter, *q_± σ _*= *± t_c_/*Г_∓*σ*_, describes the degree of electron phase coherence between two different paths. For example, one is the path through the bonding molecular state, and the other is the path through the antibonding molecular state. Г*_± σ _*is the expanding function due to the coupling between the bonding (antibonding) molecular state and metallic electrodes, which is given by Γ±σ=Γ0±Γ0 cos(ϕσ2). When the spin-dependent electron phase is considered, the transmission spectrum is composed of four resonant peaks, and their asymmetrical degrees can thus be marked by the parameter *q_± σ_*. In the absence of the interdot tunnel coupling (*t_c _*= 0), a symmetrical transmission node (*q_± σ _*= 0) arising from the quantum destructive interference is obtained. In the presence of the interdot tunnel coupling (*t_c _*= Г_0_) and absence of the magnetic flux (*ϕ *= 0), the relation between the spin-up and spin-down phase factors owns *ϕ_↑ _*= *-ϕ_↓_*. The transmission probability *τ_σ_*, Seebeck coefficient *S_σ _*and Lorenz number *L_σ _*become spin-independent as shown in Figure 2), 1), and 1), respectively. In this case, the transmission probability *τ_σ _*as a function of the chemical potential displays a near symmetrical Breit-Wigner peak centered at the bonding molecular state and an asymmetrical Fano line shape centered at the antibonding molecular state. The degree of the asymmetry of the Fano-Like peak can be attributed to the electron phase coherence. In the table 1, we calculate the approximate values of *q_± σ _*of four resonate peaks for different AB phase *ϕ *with *ϕ_R _*= 0.5*π*. For *ϕ *= 0, we find *q*_+*↑ *_= *q*_+*↓*_≃ 6.8 (near symmetrical Breit-Wigner peak at energy -*t_c_*) and *q*_-*↑ *_= *q*_-*↓*_≃ -1.2 (Fano-Like peak at energy *t_c_*). According to Eq. (12), an asymmetrical transmission node centered at energy *t_c_/cos*(*ϕ_R_/*2) can be found as shown in Figure 2 (a1). So we find that Seebeck coefficient *S_↑ _*= *S_↓ _*is enhanced strongly in the vicinity of the asymmetrical transmission node, and the corresponding value of Lorenz number *L_↑ _*= *L_↓ _*in units of *L_WF _*at the asymmetrical transmission node approaches to a temperature-independent value of 4.2 [55]. Once the AB phase *ϕ *is presented, the asymmetrical transmission node splits into two spin-dependent asymmetrical transmission nodes at energies *t_c_/cos*(*ϕ_σ_/*2). *S_↑ _*and *L_↑ _*are enhanced strongly in the vicinity of energy *t_c_/cos*(*ϕ_↑_/*2), and *S_↓ _*and *L_↓ _*are enhanced strongly in the vicinity of energy *t_c_/cos*(*ϕ_↓_/*2). Some interesting features in table 1 and Figure 2 should be noted as the following expressions. First, *q_± σ _*has a negative value when the spin-dependent molecular states are located at the high energy region, while *q_± σ _*has a positive value when they are located at the low energy region. We also find that the region of the enhanced thermoelectric effects appears at the molecular states with the lower value of *|q_± σ_|*. For example, when *ϕ *= 0.25*π *and *ϕ_R _*= 0.5*π*, *S_↑ _*is enhanced strongly in the vicinity of the molecular states with *q*_-*↑ *_= -1.0, and *S_↓ _*can be enhanced strongly in the vicinity of the molecular states with *q*_-*↓ *_= -1.4. Second, *S_σ _*always has a larger positive value when *q_± σ _<*0, and *S_σ _*has a smaller negative value when *q_± σ _>*0. The last feature is that one spin component of Seebeck effects can be tuned while the other spin component is retained. The behind reason is that the behavior of the spin-dependent transmission as a function of the chemical potential is dominated by the level expanding functions Г*_± σ_*, which gives rise to a similar behavior of the Seebeck effects as a function of the chemical potential.

**Table 1:** Approximate values of *q_± σ _*for various different values of *ϕ*

*ϕ*	*q*_+*↑*_	*q*_+*↓*_	*q_-↑_*	*q_-↓_*
0	6.8	6.8	-1.2	-1.2

0.25*π*	26.3	3.2	-1.0	-1.4

0.75*π*	26.3	1.4	-1.0	-3.2

1.25*π*	3.2	1.0	-1.4	-26.3

1.75*π*	1.4	1.0	-3.2	-26.3

2.25*π*	1.0	1.4	-26.3	-3.2

**Figure 3:**
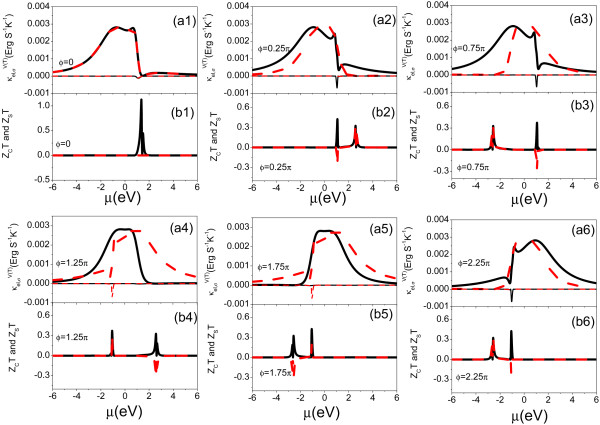
**(Color online) Spin-dependent electronic thermal conductance $\kappa_{el,\sigma }^{V}$ and $\kappa_{el,\sigma }^{T}$, charge FOM *Z_C_T *and spin FOM *Z_S_T *as function of the chemical potential *μ *under several different values of *ϕ *at room temperature (T = 300 K)**. Thick black solid (red dashed) lines in [*an*(*n *= 1,..., 6)] denotes spin-up electronic thermal conductance $\kappa_{el,↑}^{T}$. Thin black solid (red dashed) lines in [*an*(*n *= 1,..., 6)] denotes spin-down electronic thermal conductance $\kappa_{el,↓}^{T}$. The black solid lines in [*bn*(*n *= 1,..., 6)] represent the charge FOM *Z_C_T*, and the red dashed lines in [*bn*(*n *= 1,..., 6)] represent the spin FOM.

In Figure 3, we calculate $\kappa_{el,\sigma }^{V\left(T\right)}$, *Z_C_T *and *Z_S_T *as functions of the chemical potential for the different values of *ϕ*. The results show that $\kappa_{el,\sigma }^{T}$ and *τ_σ _*has a similar behavior due to $\kappa_{el,\sigma }^{T}\propto \tau_{\sigma }$ in the lower temperature region. $\kappa_{el,\sigma }^{V}$ has a negative value for the whole energy region due to $\kappa_{el,\sigma }^{V}\infty -{\left(\tau^{\prime }\left(\mu \right)\right)}^{2}$, and it should be noted that $\kappa_{el,\sigma }^{V}$ has an obvious negative value in the vicinity of transmission peak with *|q_± σ_| *≃ 1.0 as shown in Figure 3 (a2), (a3), (a4), (a5), and (a6). *Z_C_T *and *|Z_S_T| *are enhanced strongly in the vicinity of transmission peaks with *|q_± σ_| *≃ 1.0 and *|q_± σ_| *≃ 1.4. The magnitude of *|Z_S_T| *can approach to that of *Z_C_T *in the vicinity of transmission peaks with *|q_± σ_| *≃ 1.4. The results indicate that a near pure spin thermoelectric generator can be obtained by tuning the AB phase *ϕ *with a fixed value of *ϕ_R_*.

**Figure 4:**
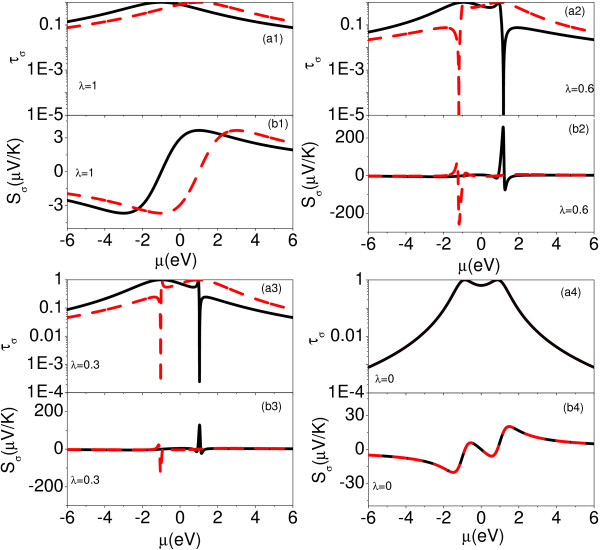
**(Color online) Spin-dependent transmission probability *τ_σ_*(logarithmic scale) and spin-dependent Seebeck coefficient *S_σ _*as functions of the chemical potential *μ *in the presence of different values of *λ *at room temperature (T = 300 K)**. *ϕ_R _*and *ϕ *have same values as *ϕ_R _*= *ϕ *= *π*. The black solid lines represents the spin-up component, and the red dashed lines represents the spin-down component.

A detail study of the spin-dependent thermoelectric effects is presented in Figure 4 when the configuration of the AB interferometer evolves from a symmetrical parallel geometry to a series. The AB phase *ϕ *and *ϕ_R _*are chosen an identical value *ϕ *= *ϕ_R _*= *π*. The spin-dependent transmission probability *τ_σ _*has the following expression as,

(14) $$\tau_{\sigma }=\frac{{\left[\frac{1+\lambda }{2}t_{c}\mp \sqrt{\lambda }\mu \right]}^{2}}{\Omega \left(\mu \right)},$$

where $\Omega \left(\mu \right)={\left[\frac{\mu^{2}-t_{c}^{2}}{2\Gamma_{0}}-\frac{{\left(1-\lambda \right)}^{2}\Gamma_{0}}{8}\right]}^{2}+{\left[\frac{1+\lambda }{2}\mu \mp \sqrt{\lambda }t_{c}\right]}^{2}$. When *λ *= 1, we have a simple expression for *τ_σ _*as,

(15) $$\tau_{\sigma }=\frac{4\Gamma_{0}^{2}}{{\left(\mu \pm t_{c}\right)}^{2}+4\Gamma_{0}^{2}},$$

where + for spin up and - for spin down. Eq. (15) shows the symmetrical spin-dependent Breit-Wigner peaks centered at *± t_c _*as shown in Figure 4). The corresponding *q_-↑ _*and *q*_+*↓ *_become infinity (see table 2). When *λ *= 0, the two QDs in a serial configuration are sandwiched between two metallic electrodes, in the case, the linear transmission probability become spin-independent due to the absence of the AB phase. The transmission probability can be calculated by the following expression,

**Table 2:** Approximate values of *q_± σ _*for various different values of *λ*

*λ*	*q*_+*↑*_	*q*_+*↓*_	*q_-↑_*	*q_-↓_*
1	+∞	No	No	-∞

0.6	78.7	1.3	-1.3	-78.7

0.3	19.6	1.7	-1.7	-19.6

0	4	4	-4	-4

(16) $$\tau_{↑}=\tau_{↓}=\frac{t_{c}^{2}\Gamma_{0}}{{\left(\mu^{2}-t_{c}^{2}-\frac{\Gamma_{0}^{2}}{4}\right)}^{2}+\mu^{2}\Gamma_{0}^{2}}.$$

**Figure 5:**
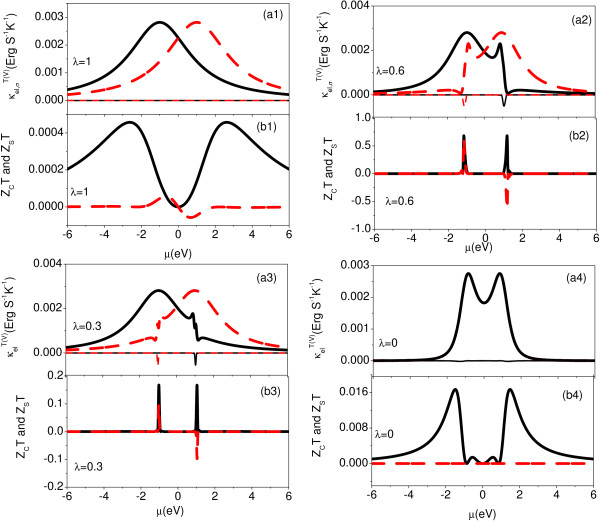
**(Color online) Spin-dependent electronic thermal conductance $\kappa_{el,\sigma }^{V}$ and $\kappa_{el,\sigma }^{T}$, charge and spin figure of merit *Z_C_T *and *Z_S_T *as function of the chemical potential *μ *under several different values of *λ *at room temperature (T = 300 K)**. Thick black solid (red dashed) lines in [*an*(*n *= 1,..., 4)] denotes spin-up electronic thermal conductance $\kappa_{el,↑}^{T}$. Thin black solid (red dashed) lines in [*an*(*n *= 1,..., 4)] denotes spin-down electronic thermal conductance $\kappa_{el,↓}^{T}$. The black solid lines in [*bn*(*n *= 1,..., 4)] represent the charge FOM *Z_C_T*, and the red dashed lines in [*bn*(*n *= 1,..., 4)] represent the spin FOM.

We note that the transmission probability vanishes when *t_c _*= 0, which means the full reflection for electrons happening in this AB interferometer. When 0 *< λ <*1, the spin-dependent transmission probability *τ_σ _*is composed of near Breit-Wigner peak and Fano line shapes as shown in Figure 4 and 3. The spin-dependent transmission probability can be approximated by,

(17) $$\tau_{\sigma }≃\tau_{+\sigma }\left(\mu \right)+\tau_{-\sigma }\left(\mu \right),$$

where $\tau_{-\sigma }\left(\mu \right)=\frac{1}{1+q_{-\sigma }^{2}}\frac{{\left[\left(\mu -t_{c}\right)+q_{-\sigma }\Gamma_{-\sigma }\right]}^{2}}{{\left(\mu -t_{c}\right)}^{2}+\Gamma_{-\sigma }^{2}}$ and $\tau_{+\sigma }\left(\mu \right)=\frac{1}{1+q_{+\sigma }^{2}}\frac{{\left[\left(\mu +t_{c}\right)+q_{+\sigma }\Gamma_{+\sigma }\right]}^{2}}{{\left(\mu +t_{c}\right)}^{2}+\Gamma_{+\sigma }^{2}}$ with $\Gamma_{\pm \sigma }=\left(1\;+\lambda \right)\Gamma_{0}∕2\pm \sqrt{\lambda }\Gamma_{0}$. From Eq. (14), we can see clearly that there are two asymmetrical transmission nodes centered at,

(18) $$\mu =\pm \frac{1+\lambda }{2\sqrt{\lambda }}t_{c},$$

where + means spin up case and - represents spin-down case. As a result, we find that the spin-dependent Seebeck effect is enhanced strongly in the vicinity the spin-dependent transmission nodes. The electronic thermal conductance $\kappa_{el}^{V\left(T\right)}$, *Z_C_T *and *Z_S_T *as functions of the chemical potential under different values of *λ *are displayed in Figure 5. $\kappa_{el,\sigma }^{T}$ has a similar behavior with the transmission probability as the chemical potential changes. $\kappa_{el,\sigma }^{V}$ has an obvious negative values in the vicinity of the spin-dependent transmission node. Similarly, *Z_C_T *and *Z_S_T *are enhanced strongly in the vicinities of the transmission nodes. As *λ *increases from 0 to 1, we find the maximum values of *Z_C_T *and *Z_S_T *become larger. The corresponding *q*_+*↓ *_and *|q_-↑_| *decrease, while *q*_+*↑ *_and *|q_-↓_| *increase as *λ *increases (see table 2).

## Summary

We investigate the spin-dependent thermoelectric effects of parallel-coupled DQDs embedded in an AB interferometer in which the RSOI is considered by introducing a spin-dependent phase factor in the linewidth matrix elements. Due to the interplay between the quantum destructive interference and RSOI in the QDs, an asymmetrical transmission node can be observed in the transmission spectrum in the absence of the RSOI. Using an inversion asymmetrical interface electric field, we can induce the RSOI in the QDs. We find that the asymmetrical transmission node splits into two spin-dependent asymmetrical transmission nodes in the transmission spectrum, which induces that the spin-dependent Seebeck effects are enhanced strongly at different energy regimes. We also examine the evolution of spin-dependent Seebeck effects from a symmetrical parallel geometry to a configuration in series. The asymmetrical couplings between the QDs and metallic electrodes induce the enhancement of spin-dependent Seebeck effects in the vicinity of the corresponding spin-dependent asymmetric transmission node in the transmission spectrum.

## Abbreviations

2DEG: two-dimensional electron gas; AB: Aharonov-Bohm; FOMs: figure-of-merits; QD: quantum dot; RSOI: Rashba spin-orbit interaction.

## Competing interests

The authors declare that they have no competing interests.

## Authors' contributions

All authors read and approved the final manuscript.
